# Development of a Gene-Based Prediction Model for Recurrence of Colorectal Cancer Using an Ensemble Learning Algorithm

**DOI:** 10.3389/fonc.2021.631056

**Published:** 2021-02-22

**Authors:** Han-Ching Chan, Amrita Chattopadhyay, Eric Y. Chuang, Tzu-Pin Lu

**Affiliations:** ^1^Department of Public Health, College of Public Health, National Taiwan University, Institute of Epidemiology and Preventive Medicine, Taipei, Taiwan; ^2^Bioinformatics and Biostatistics Core, Center of Genomic and Precision Medicine, National Taiwan University, Taipei, Taiwan; ^3^Department of Electrical Engineering, Graduate Institute of Biomedical Electronics and Bioinformatics, National Taiwan University, Taipei, Taiwan

**Keywords:** colorectal cancer, machine learning, gene expression, prognostic signature, ensemble

## Abstract

It is difficult to determine which patients with stage I and II colorectal cancer are at high risk of recurrence, qualifying them to undergo adjuvant chemotherapy. In this study, we aimed to determine a gene signature using gene expression data that could successfully identify high risk of recurrence among stage I and II colorectal cancer patients. First, a synthetic minority oversampling technique was used to address the problem of imbalanced data due to rare recurrence events. We then applied a sequential workflow of three methods (significance analysis of microarrays, logistic regression, and recursive feature elimination) to identify genes differentially expressed between patients with and without recurrence. To stabilize the prediction algorithm, we repeated the above processes on 10 subsets by bagging the training data set and then used support vector machine methods to construct the prediction models. The final predictions were determined by majority voting. The 10 models, using 51 differentially expressed genes, successfully predicted a high risk of recurrence within 3 years in the training data set, with a sensitivity of 91.18%. For the validation data sets, the sensitivity of the prediction with samples from two other countries was 80.00% and 91.67%. These prediction models can potentially function as a tool to decide if adjuvant chemotherapy should be administered after surgery for patients with stage I and II colorectal cancer.

## Introduction

Colorectal cancer (CRC) is one of the most commonly occurring cancers worldwide (1). In Taiwan, colorectal cancer was the second leading incident cancer in 2016 (2). Currently, surgery is considered the primary treatment for CRC patients, followed by optional adjuvant chemotherapy to decrease the risk of metastasis and local recurrence. The decision of whether to use adjuvant chemotherapy is based on clinical factors such as the American Joint Committee on Cancer (AJCC) staging system (3). However, it is still controversial whether adjuvant chemotherapy should be administered to stage I and II CRC patients. According to clinical trials to date, the benefits of adjuvant chemotherapy for stage II CRC patients were inconsistent and minor; that is, the benefits have failed to attain statistical significance (4, 5). Therefore, considering the adverse effects and tremendous direct and indirect costs, whether adjuvant chemotherapy should be offered to all stage II CRC patients deserves further investigation (6).

Based on evidence from a nationwide cohort study in the United States, adjuvant chemotherapy has been more frequently given to younger patients (7). However, the survival rate of the younger patients did not significantly differ from that of their older counterparts who did not undergo adjuvant chemotherapy, suggesting that they did not necessarily require adjuvant chemotherapy. Moreover, there is considerable cost associated with such unnecessary treatments. For example, in Taiwan, the total medical expenses for colorectal cancer were about 33 million dollars (USD) in 2016, which accounted for 13.4% of all cancer medical expenses (8).

Although adjuvant chemotherapy is not routinely recommended for stage II patients, according to the Cancer Registry Annual Report (2016) of Taiwan, the rate of surgery with adjuvant chemotherapy in stage II patients reached 53.46% (2). If patients who genuinely need intensive treatment to prevent a recurrence could be successfully identified, it would not only prevent patients from suffering the side effects of unnecessary treatment protocols but would also reduce unnecessary healthcare costs. The American Society of Clinical Oncology (ASCO) guidelines indicate that adjuvant chemotherapy should only be recommended for some “high risk” stage II patients as opposed to a routine recommendation for all stage II patients (9). Though several clinical characteristics have been suggested to impart a high risk of recurrence, such as lymphovascular invasion, T4 primary tumors, poor differentiation of tumors, and bowel perforation and/or obstruction, a well-defined list of factors that predict recurrence is still lacking (10). Thus, a reliable method is needed to identify stage I and II patients with high risk of recurrence.

Microarray gene expression profiling is a widely used tool to determine the prognosis of cancer, including breast cancer (11), non-small cell lung cancer (12), prostate cancer (13), and others (14). A successfully developed genetic test called MammaPrint was approved by the US Food and Drug Administration (FDA) to predict the risk of recurrence in stage I and II breast cancer patients (15, 16). Over the past decade, several prognostic biomarkers from microarray gene expression profiling have been identified in CRC, using widely used gene profiling assays (17, 18). Although these assays have improved the classification of patients with high risk of recurrence or survival, none of them were able to be incorporated with current guidelines regarding the recommendation of adjuvant chemotherapy. Therefore, a more helpful and robust gene signature needs to be determined. In this study, we aimed to determine a gene signature using public gene expression data that could successfully identify high risk of recurrence among stage I and II CRC patients.

## Materials and Methods

**Figure 1:**
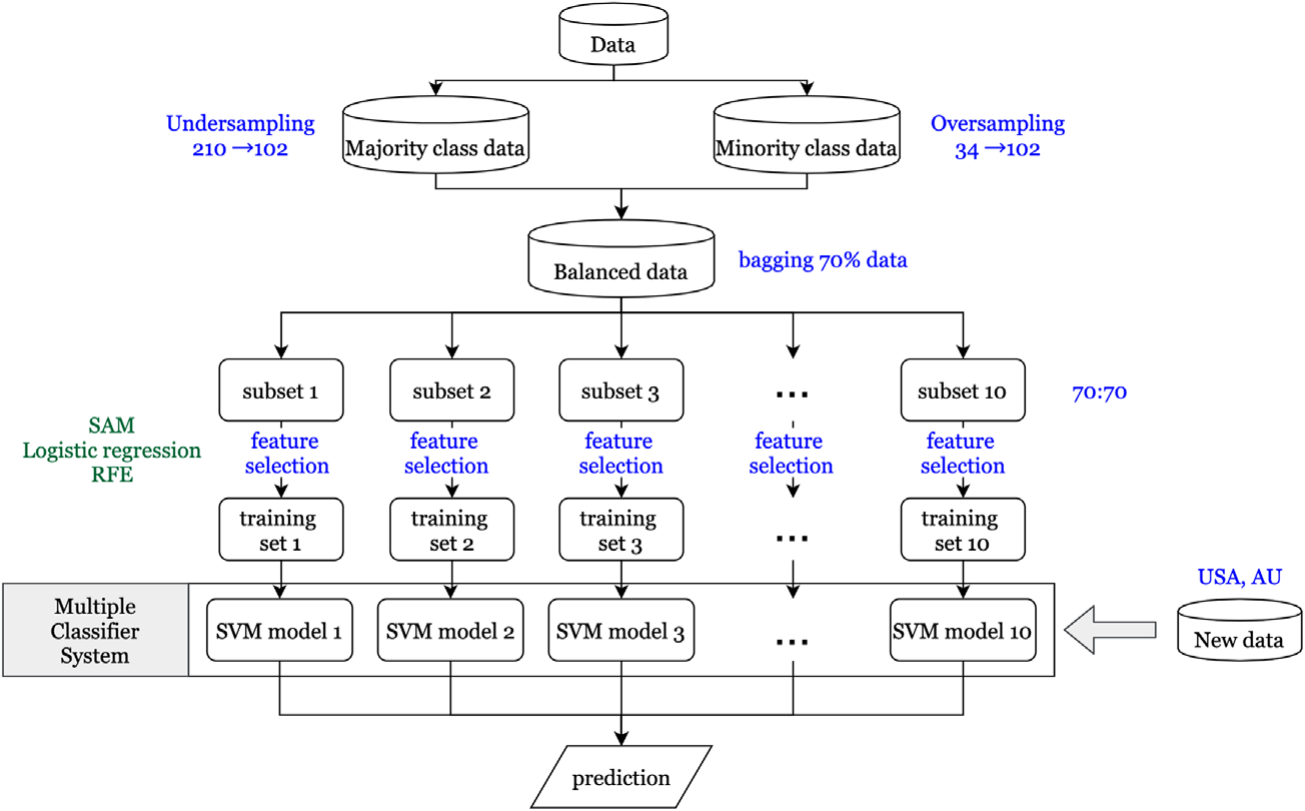
Flowchart for data analysis.

An overview of the workflow implemented in this study is shown in **Figure 1**. It gives a comprehensive view of the data sets used and the various techniques and methodologies applied.

### Data Sets

All microarray data sets analyzed in this study (**Table 1**) were retrieved from public domains, including GSE40967, GSE17536, and GSE14333 from the Gene Expression Omnibus (GEO) (https://www.ncbi.nlm.nih.gov/geo/), which were obtained using the Affymetrix HG-U133 Plus2.0 Chip microarray platform. The reasons why we selected these three microarray data sets were that all these data sets were analyzed by the same microarray platform and reported the recurrence status. All raw data as CEL files were normalized with robust multichip averaging using the “affy” package of R software (22) and subsequently processed by quantile normalization. Among these data sets, GSE40967 from France was used as the training data set to identify prognostic biomarkers and develop the prediction models. The other two data sets from the USA (GSE17536) and Australia (GSE14333) were utilized as the testing data to validate the performance of the prediction model. GSE40967 consisted of 750 stage I to IV colon cancer patients who underwent surgery between 1987 and 2007; the data included each patient’s recurrence status and date of death, if applicable. Out of these patients, 196 with stage I or II who did not receive any adjuvant chemotherapy after surgery were used for our analyses. The primary outcome was recurrence-free survival, and the definition of recurrence was annotation of a recurrence in the data set within three years after undergoing the surgery.

**Table 1:** Characteristics of three public gene expression data sets.

Data Set	Year	Country	Sample (used)	Adjuvantchemotherapy	Reference
GSE40967	2012	France	750 (196)	Yes	(19)
GSE17536	2009	USA	177 (55)	NA	(20)
GSE14333	2009	Australia	290 (103)	Yes	(21)

### Imbalanced Data

A vital issue in the machine learning field is that the classifier using imbalanced data tends to be biased in predicting the majority class. Therefore, the synthetic minority over sampling technique (SMOTE) (23) is used to balance the proportions of the majority class (no recurrence) and the minority class (recurrence). For generating synthetic samples, SMOTE calculates the k nearest neighbors for each minor class sample and randomly chooses one or more of the k nearest neighbors depending on the amount of oversampling samples needed for each minor class sample. Consequently, the synthetic samples are created randomly, along with the line connection with one or more k nearest neighbors. Oversampling the minority class might generate too many synthetic samples, which would lead to data overfitting. To prevent this situation, oversampling of minority and undersampling of majority class techniques were simultaneously applied to generate new samples. In this study, we have included rare recurrence events (n = 34; 17.3%) for stage I and II patients, based on the recurrence rate in the 196 patients from data set GSE40967. Oversampling from the minority class of 34 patients generated 102 synthetic samples, and undersampling from the majority class of 162 generated 102 samples.

### Feature Selection

First, to identify differentially expressed genes, three statistical methods (significance analysis of microarrays (SAM), logistic regression, and recursive feature elimination (RFE)) were used. Each statistical method depends on different characteristics of the data, so the genes that pass the thresholds for all three methods are assumed to have a more significant influence on CRC recurrence than other genes. SAM uses a modified t-statistic to evaluate the differential expression of each gene between real data and randomly permutated data (24). Univariate logistic regression analysis is performed on each gene that passed through SAM to estimate its effect on recurrence. The ranked coefficients of logistic regression are then plotted to determine the cut point of the threshold by the knee of the curve of coefficients plot. Finally, RFE with a random forest method is applied to determine differentially expressed genes (25). The basic idea of RFE is to find the minimal set of variables resulting in an excellent prediction performance by recursively running random forests as well as removing a specified proportion of least important variables until the variable set converges or the time of the loop is done (26). Therefore, the minimum set of variables obtained from RFE is our final set of significantly differentially expressed genes.

### Parallel Ensemble Method

Certain features of the data might have a significant impact on our resulting set of differentially expressed genes and their subsequent validation performance. One feature is the minority class, consisting of patients with recurrence within three years. As previously mentioned, the new synthetic minority class samples that were generated by SMOTE to obtain balanced data might contribute to the prediction model, and even dominate the results if the proportion of synthetic samples is too large (27). Second, RFE *via* a random forest method is a convergence-based algorithm method; hence, the final set of significantly differentially expressed genes would be slightly different each time. Therefore, to get a more stable prediction performance, the ensemble method is used to determine a set of classifiers that make the final prediction (28). First, 10 subsets are generated using the bagging technique (29) to randomly extract about 70% of the study subjects from the balanced data in each iteration. This prevents the synthetic samples from dominating the results, as their proportion would not be overwhelmingly more than that of real samples every time. Next, for each subset, the same feature selection processes are conducted to obtain the significantly differentially expressed gene sets. Then each gene set is used to construct the prediction models using a support vector machine (SVM) method (30). Finally, since different models might predict different results for the same patient, the majority voting method is applied to determine the final prediction for each patient. Furthermore, it is more important to predict high risk patients correctly compared to low risk patients in our study, so the F_2_ score, which expresses both the precision and recall of the prediction, is used as another evaluation of prediction performance.

### Effective Drug Prediction

In addition to the prediction of recurrence risk in CRC patients, we also tried to identify suitable drugs for the different risk groups. The drug response results were based on data set GSE36133 (31), which was originally from the Cancer Cell Line Encyclopedia (CCLE) and aimed to establish the association between drugs and genes by investigating the response to 24 different drugs in a variety of cancer cell lines. In the CCLE data set, only 22 cell lines belonged to CRC and thus we focused on them to perform further investigations. Also, the expression of these 22 cell lines was detected using the same microarray platform as mentioned above. Our prediction model was applied to predict the risk of recurrence and then determine which drug elicits a significantly different response between the high risk and low risk groups in order to identify potential therapy targets.

### Other Methods for Comparison of Prediction Performance

To check whether or not the general feature selection method could work, we also used lasso (32) and logistic regression methods. Logistic regression with forward selection was applied for each subset. The cut points for the probability of prediction for the 10 SVM models were separately determined by receiver operating characteristic curve analysis. Regarding the lasso method, the value of lambda was determined by cross-validation. We used sensitivity, specificity, and F_2_ scores as the performance indicators to evaluate these models.

## Results

### Clinical Feature Analysis

The 196 patients from France who did not undergo adjuvant chemotherapy after surgery were split into two groups based on whether their cancer recurred within three years. The 3-year recurrence rate in stage I and II CRC patients was 17.3% (34/196, **Table 2**). In this study, most of the clinical features, including age, gender, and mutation of *TP53*, *KRAS*, or *BRAF*, did not attain a statistically significant difference between the recurrent and non-recurrent groups (**Table 2**). The only feature that nearly reached statistical significance for the difference between the recurrent and non-recurrent groups was the cancer stage (Fisher’s exact test p-value = 0.0548), with recurrence rates of 3.6% in stage I and 19.6% in stage II.

**Table 2:** Clinical feature analysis.

GSE40967 N = 196		RecurrenceN = 34 No. (%)^a^	Non-recurrenceN = 162 No. (%)	P-value
Age, mean (SD)		71·79 (12·7)	68·57 (12.6)	0·1854
Stage	I	1 (3·6)	27 (96·4)	0·0548
	II	33 (19·6)	135 (80·4)	
Gender	Male	22 (20·4)	86 (79·6)	0·2572
	Female	12 (13·6)	76 (86·4)	
TP53	M	15 (26·8)	41 (73·2)	0·3877
	WT	12 (19·7)	49 (80·3)	
KRAS	M	12 (18·8)	52 (81·2)	0·8394
	WT	20 (17·1)	97 (82·9)	
BRAF	M	4 (25·0)	12 (75·0)	0·3144
	WT	25 (15·9)	132 (84·1)	

M, mutation; SD, standard deviation.

^a^All values are presented as number (%) unless otherwise indicated.

### Determination of Differentially Expressed Genes From Feature Selection

After hybrid data resampling using SMOTE, sample sizes of both the majority (no recurrence) and minority (recurrence) class were adjusted to 102. In the 10 subsets, the mean number of differentially expressed candidate genes that passed the SAM threshold (delta≧0.6) were 13,285, of which 1,417 candidate genes also passed the univariate logistic regression threshold (coefficient≧2.4). Finally, after passing through random forest RFE, the mean number of significantly differentially expressed genes was 11. The total number of unique differentially expressed genes in the 10 subsets was 51.

### Prediction of 3-Year Recurrence-Free Survival Using Gene Expression Data

**Figure 2:**
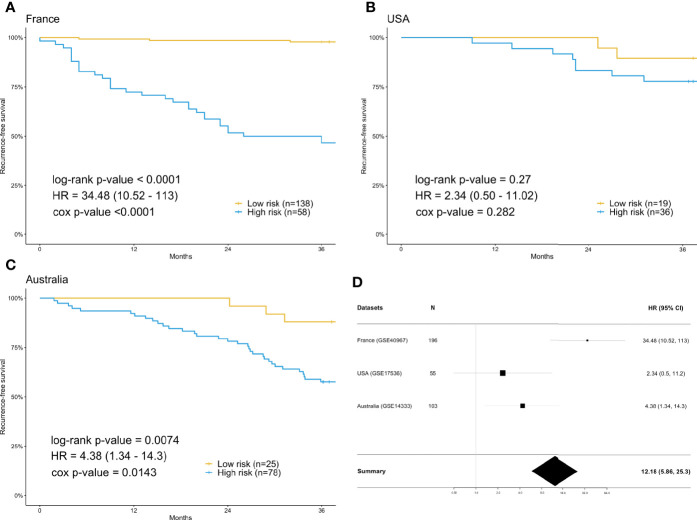
Survival analysis using the training data set (France) and validation data sets (USA & Australia). **(A)** Kaplan-Meier plot for France (n=196) data set. **(B)** Kaplan-Meier plot for USA (n=55) data set. **(C)** Kaplan-Meier plot for Australia (n=103) data set. **(D)** Forest plot of the hazard ratio and 95% confidence intervals in both the training data set and validation data sets. The prediction of high or low risk groups was dependent on majority voting. The P-values correspond to the two-sided log-rank test determining the difference between two curves.

For each differentially expressed gene set, the prediction model was constructed using SVM with the polynomial kernel, as it had the best explanation compared with other kernels. For the determination of the final prediction, the majority voting was set to 7, which means that only if 7 or more of the 10 models predicted the patients would recur in 3 years would the patients be classified as a high-risk group for recurrence. The Kaplan-Meier survival plot (**Figure 2A**) shows that the classification result is significantly associated with the recurrence-free survival time for the France data set (log-rank test p-value <0.0001, data used here was real data before hybrid resampling). The sensitivity, specificity, and the F_2_ score of the voting prediction were 91.18%, 83.33%, and 89.49%, respectively (**Table 3**). These results showed that in our prediction, the patients who were classified as low risk had a much better prognosis than those classified as high risk.

**Table 3:** The comparison of the prediction performance of different methods.

	SAM+LR+RFE			LR with forward selection			Lasso		
	Training^a^	USA	AU	Training	USA	AU	Training	USA	AU
Accuracy	0·8469	0·4545	0·534	0·7347	0·7636	0·6505	0·8724	0·6182	0·6019
Sensitivity	0·9118	0·8	0·9167	0·7059	0·1	0·1667	0·9412	0·4	0·3889
Specificity	0·8333	0·3778	0·3284	0·7407	0·9111	0·9104	0·8580	0·6667	0·7164
F_2_ score	0·8949	0·6539	0·6749	0·7126	0·1217	0·1993	0·9233	0·4348	0·4280

AU, Australia; LR, logistic regression; SAM, significance analysis of microarrays; RFE, recursive feature elimination.

^a^The training data set was from France.

### Prediction Performance in the Validation Data Sets From the USA and Australia

To check the accuracy of our proposed prediction model, we used two independent validation data sets, gene expression data from the USA (n=177) and Australia (n=290). Similar inclusion criteria and data preprocessing to that of the training data set were applied. The final sample sizes were 55 patients from the USA and 103 from Australia. The Kaplan-Meier survival plots in **Figures 2B, C** show that the prediction model could successfully separate the CRC patients at high and low risk for recurrence-free survival. The p-values of the log-rank test for the USA and Australia data sets were 0.27 and 0.0074, respectively. However, the insignificant p-value for the USA sample might be due to the small sample size. The sensitivity, specificity, and F_2_ score of the model in the USA data set were 80.00%, 37.78%, and 65.39%, respectively, while those in Australia data set were 91.67%, 32.84%, and 67.49%, respectively (**Table 3**). We also estimated the hazard ratio using a Cox proportional hazards model (**Figure 2D**). The hazard ratios for the USA and Australia data sets were 2.34 (0.5, 11.2) and 4.38 (1.34, 14.3), respectively. Additionally, the overall summary estimate of all data sets was 12.18 (5.86, 25.3).

### Prediction of Drug Response

For each of the 24 drugs, we applied the Wilcoxon rank sum test to determine significantly different drug responses, as the sample size did not fit the normal distribution. Among the 24 drugs, no p-value passed the threshold of 0.05 (**Table 4**). The most significant one was AZD6244 (p-value=0.0982), an investigational MEK inhibitor which has been found to elicit a promising response in CRC patients with high risk of recurrence. The prediction of the effects of drug use needs further investigation and validation.

**Table 4:** The statistical results for 24 anti-cancer compounds using the Wilcoxon rank sum test.

Compound	P-value	Compound	P-value	Compound	P-value	Compound	P-value
AZD6244	0·0982	PD-0332991	0·3352	Lapatinib	0·5936	PHA-665752	0·8983
PD-0325901	0·1662	Irinotecan	0·4103	L-685458	0·6218	Nutlin-3	0·9671
ZD-6474	0·2622	Paclitaxel	0·4845	Erlotinib	0·6396	TKI258	0·9671
AEW541	0·3002	RAF265	0·5381	LBW242	0·6521	AZD0530	1
Panobinostat	0·3002	Sorafenib	0·5381	Topotecan	0·7120	PF2341066	1
17-AAG	0·3168	PLX4720	0·780	Nilotinib	0·8096	TAE684	1

### Comparison of the Prediction Performance With Other Methods

The comparison of results from different methods is shown in **Table 3**. For logistic regression with forward selection, the sensitivity, specificity, and F_2_ score of the model in the training data set were 70.59%, 74.07%, and 71.26%, respectively, while those in the validation data sets were 10.00%, 91.11%, and 12.17% in the USA data set and 16.67%, 91.04%, and 19.93% in Australia data set. For the lasso method, the sensitivity, specificity, and F_2_ score in the training data set were 94.12%, 85.80%, and 92.33%, respectively, while those in the validation data sets were 40.00%, 66.67%, and 43.48% in the USA data set and 38.89%, 71.64%, and 42.80% in Australia data set.

## Discussion

In this study, we successfully identified prognostic biomarkers to predict the risk of recurrence in stage I and II CRC patients using microarray gene expression data sets. Based on the criteria previously mentioned, we defined rare recurrence events as the primary outcome of interest. To address the problem of imbalanced data, SMOTE was used to balance the proportion between the majority class and minority class. The differentially expressed genes were passed through three statistical methods, SAM, logistic regression, and RFE, and subsequently a prediction model was constructed by SVM (24, 25, 30). Furthermore, to stabilize the performance of the results, we constructed 10 independent models, and the final prediction was decided by majority voting. The proposed prediction model was found to perform well in terms of sensitivity for both the training and validation data sets. Also, the result of overall summary hazard ratio estimate indicated that our predictors could effectively classify patients into high risk and low risk groups.

Amongst the above-mentioned series of processes, the potential impact of imbalanced data on our results posed the greatest challenge. SVM is capable of handling such data by assigning higher misclassification penalties to minority classes; however, it failed to work perfectly for our study. Therefore, the data resampling method was applied to solve this problem. The reason that we adopted the hybrid resampling rather than simple oversampling is that the latter would lead to overfitting when applied to predicting the validation data. This situation implied that the synthetic samples were over-generated, thus dominating the results. In order to control the proportion of synthetic samples, we reduced the number of synthetic samples from the minority class and undersampled the majority class. Although we lost some information on the majority class, this ensured that the proportion of synthetic samples would not be too large.

For the feature selection and model construction, we applied three statistical methods and used SVM to construct prediction models with majority voting. For comparison, we also used the logistic regression and lasso methods. The results showed that, although the performance was not too bad for the training data set, it was poor for both validation data sets. This suggests that, for high-dimensional data with a small sample size, logistic regression and lasso might not be the best choice due to their limitations, such as the finite design matrix. Thus, instead of applying a single feature selection method, we constructed multiple feature selection methods to filter out significant genes sequentially, and we used univariate logistic regression as one of the feature selection methods.

We repeated the analysis described in this study to analyze the gene expression data from stage II patients only. The sample numbers of the three analyzed data sets dropped to 168, 37, and 66, respectively, which means that around 30% of the samples were removed. Following the same analysis procedure described in this study, 130 differentially expressed genes were identified from the ten subsets. Notably, 20 of the 130 differentially expressed genes identified from stage II patients overlapped with the 51 differentially expressed genes identified from the original analysis of stage I and II patients. This significant overlap (Fisher’s exact test p-value < 0.0001) indicated that our algorithm had a robust performance. For the prediction performance, the sensitivity, specificity, and the F_2_ score were 90.91%, 91.85%, and 86.71%, respectively, in the training data set (France). For the validation data sets, the sensitivity, specificity, and F_2_ score in the USA data set (GSE17536) were 77.78%, 35.71%, and 57.38%, respectively, while those in the Australia data set (GSE14333) were 70.37%, 20.51%, and 65.07%, respectively. Due to the smaller sample size of the validation data sets, the log-rank test did not attain a value indicating significance.

**Figure 3:**
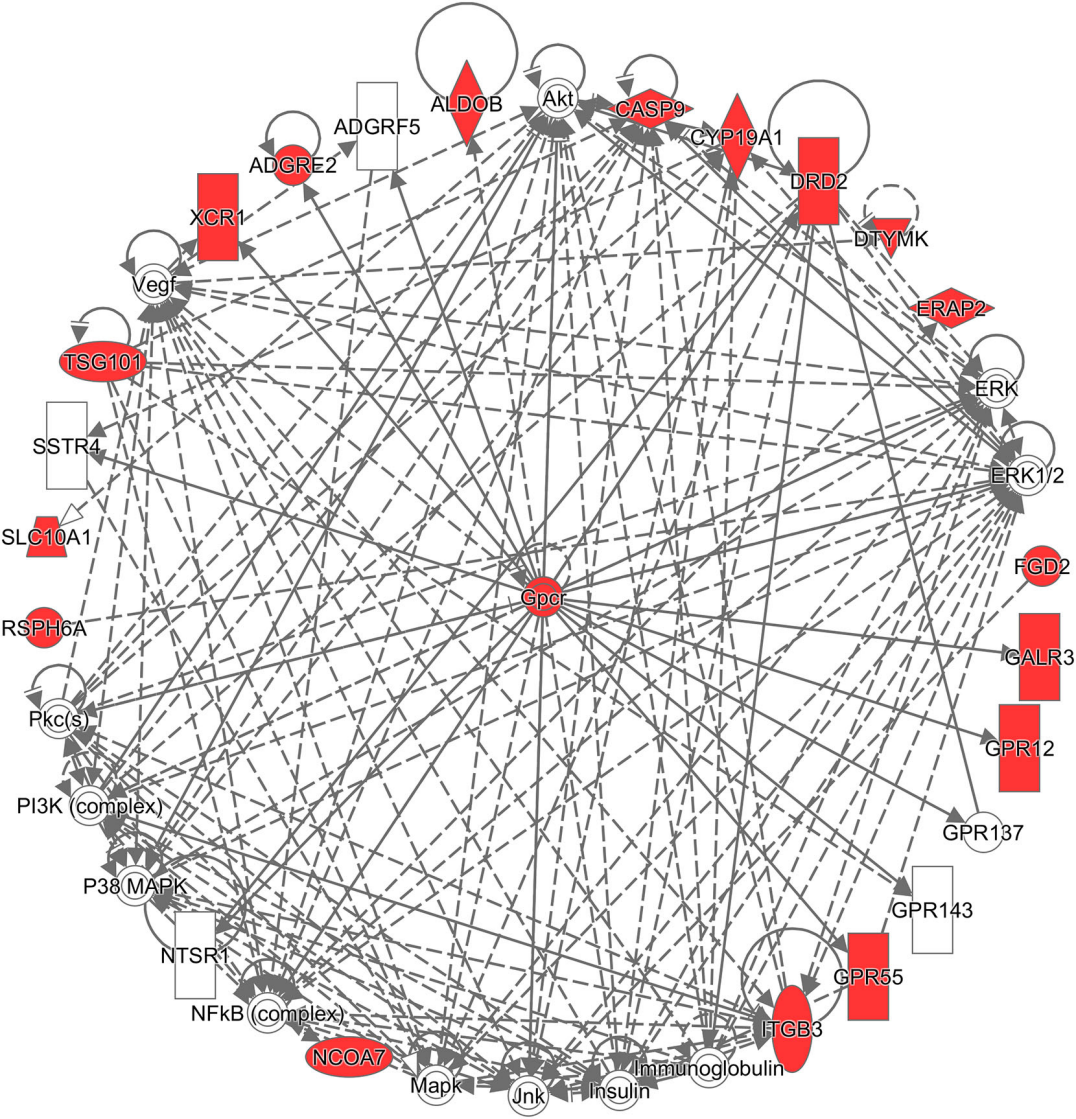
Network analysis using the Ingenuity^®^ Pathway Analysis (IPA^®^) software program. The red colored the genes which are in the list of our differentially expressed genes, and white colored the putative genes based on IPA database.

Additionally, to infer which genes among the differentially expressed genes are potentially associated with CRC, the Ingenuity^®^ Pathway Analysis (IPA^®^) software program (QIAGEN Inc., https://www.qiagenbio-informatics.com/products/ingenuity-pathway-analysis) (33) and the Database for Annotation, Visualization, and Integrated Discovery (DAVID) (34) were used. To get a comprehensive view of these CRC-related genes, an overall survival analysis was performed on the Pathology Atlas database (www.proteinatlas.org/pathology), which provides interactive survival plots using publicly available data from The Cancer Genome Atlas (35). From the functional analysis, we found that some of the differentially expressed genes belong to G-protein coupled receptors (GPCRs) (**Figure 3**), which are the largest family of cell surface receptors. In **Figure 3**, it is notable that *XCR1*, *ADGRE2*, *DRD2*, *GALR3*, *GPR12*, and *GPR55* had direct interactions with GPCRs. The top two functions with a significant p-value and more than 10 molecules were “Nonhematologic malignant neoplasm” and “Communication of cells.” These functions may have an association with CRC prognosis. A previous study reported that some mutations in the *DRD2* gene were associated with colorectal cancer (36). *GPR55* is up-regulated in CRC tumor tissue, and such alteration was reported to lead to changes in immune cells (37). Regarding the analysis done by the DAVID website, *DRD2, CYP19A1, CASP9*, and *ITGB3* were found to be associated with CRC (36, 38–43). For example, the mRNA expression of *CASP9* was down-regulated in tumor tissue compared to marginal tissue, and *ITGB3*, involved in reactive oxygen species-induced migration and invasion processes, is known to be a malignant indicator in CRC. In a comparison of our study with the survival analysis performed on the Pathology Atlas database, *ADGRE2*, *GALR3, DRD2*, and *CYP19A1* consistently displayed a trend of up-regulation in the group of CRC patients with poor prognosis.

A limitation of this study is that the specificity of the prediction for the validation data sets was found to be slightly low, which seems consistent with other previous studies (44–49). However, our model could successfully predict 30–40% of low-risk recurrence in patients, which might save many healthcare costs. Given that the healthcare cost of chemotherapy is about 22,000 dollars (USD) for stage I and II colon cancer patients, according to NICE technology appraisals, the low risk of recurrence that our model is capable of predicting could potentially save about 44 million dollars (USD) (41,700 CRC patients * 0.44 stage I and II * 0.31 received chemotherapy * $22,000 chemotherapy cost * 0.35 low-risk patients) in the UK per year (50, 51). To validate and improve our model in the future, a larger sample size would be needed.

In this study, our prediction model was developed based on gene expression features. To date, several prediction algorithms for prognosis and survival outcomes were developed in CRC patients using clinical variables and biochemical markers (52–54). The Colon Life nomogram consists of three clinical variables and one biochemical marker, including Primary tumor resection, ECOG performance status (ECOG PS), Peritoneal Metastasis, and lactate dehydrogenase (LDH) (53). Notably, the gene expression data analyzed in this study were derived from tumor tissues, which means that our prediction model can only predict the recurrence risk for patients who have undergone primary tumor resection. In contrast, the Colon Life nomogram can make predictions for patients with and without surgery. Furthermore, the Colon Life nomogram predicts the probability of overall survival, but our algorithm focuses on the recurrence event. Due to the lack of the three clinical variables and LDH in public genomic data sets, we cannot directly compare the Colon Life nomogram and our algorithm. Our prediction model may have a better prediction performance if it could integrate more clinical variables and other algorithms in the future.

## Data Availability Statement

Publicly available datasets were analyzed in this study. These data can be found here: Gene Expression Omnibus (https://www.ncbi.nlm.nih.gov/geo/). GSE40967, GSE17536 and GSE14333.

## Author Contributions

H-CC and T-PL conceived the project. EC and T-PL provided administration support of this project. H-CC implemented the project, carried out literature search, data analysis, and prepared all the figures. T-PL and H-CC did all data interpretation and concluded the findings. T-PL and H-CC accessed and verified the underlying data. H-CC, AC, and T-PL wrote the manuscript. All authors contributed to the article and approved the submitted version.

## Funding

This work has been supported in part by the Center of Genomic and Precision Medicine, National Taiwan University, Taiwan (106R8400) and the Center for Biotechnology, National Taiwan University, Taiwan (GTZ300).

## Conflict of Interest

The authors declare that the research was conducted in the absence of any commercial or financial relationships that could be construed as a potential conflict of interest.
